# Effects of stanozolol on normal and IL-1β-stimulated equine chondrocytes in vitro

**DOI:** 10.1186/s12917-018-1426-z

**Published:** 2018-03-20

**Authors:** Mariana Castro Martins, Mandy J. Peffers, Katie Lee, Luis M. Rubio-Martinez

**Affiliations:** 1 Department of Equine Clinical Studies, Institute of Veterinary Science, University of Liverpool, Leahurst Campus, Chester High Road, Neston, CH64 7TE UK; 2 Department of Musculoskeletal Biology, Institute of Ageing and Chronic Disease, University of Liverpool, Liverpool, UK

**Keywords:** Osteoarthritis, Stanozolol, Chondrocyte, Equine, Gene expression, In vitro

## Abstract

**Background:**

Intra-articular administration of stanozolol has shown promising results by improving the clinical management of lameness associated with naturally-occurring osteoarthritis (OA) in horses, and by decreasing osteophyte formation and subchondral bone reaction in sheep following surgically induced OA. However, there is limited evidence on the anti-inflammatory and modulatory properties of stanozolol on articular tissues. The objective of the current study was to evaluate the effects of stanozolol on chondrocyte viability and gene expression in normal equine chondrocytes and an inflammatory in vitro system of OA (interleukin-1β (IL-1β) treated chondrocytes).

**Results:**

Chondrocytes from normal metacarpophalangeal joints of skeletally mature horses were exposed to four treatment groups: (1) media only (2) media+IL*-*1β (3) media+IL*-*1β + stanozolol (4) media+stanozolol. Following exposure, chondrocyte viability and the expression of catabolic, anabolic and structural genes were determined. General linear models with Dunnet’s comparisons with Bonferroni’s adjustment were performed. Cell viability was similar in all groups. Stanozolol treatment reduced gene expression of MMP-13, MMP-1, IL-6 and COX-2 in both normal and IL*-*1β treated chondrocytes. Stanozolol treatment reduced ADAMTS4 gene expression in normal chondrocytes. Stanozolol reduced the expression of COL2A1.

**Conclusions:**

The current study demonstrates stanozolol has chondroprotective effects through downregulation of genes for pro-inflammatory/catabolic cytokines and enzymes associated with OA. However, there is no evidence of increased cartilage stimulation through upregulation of the anabolic and structural genes tested.

## Background

Osteoarthritis (OA) is caused by a combination of biomechanical and biochemical changes in the joint that include synovium and subchondral bone abnormalities, ultimately resulting in cartilage degeneration [1]. In the past decade, major advances have been made in molecular biology and OA research in both human and veterinary medicine. Equine models have been used extensively providing invaluable insights into the molecular pathogenesis during disease establishment and in response to treatment [2, 3].

One of the main goals in the treatment of OA is to inhibit further progression of cartilage degeneration and to restore a functional synovial environment with the potential to promote cartilage repair. Cartilage repair is mediated by the balance between chondrocyte gene expression of catabolic and anabolic genes [4, 5]. The most important mediator of cartilage degeneration is interleukin-1 (IL-1) [5, 6] and it has been reported that low innate production of IL-1β and IL-6 is associated with the absence of OA in old age [7]. IL-1 enhances the expression of pro-inflammatory mediators including matrix metalloproteinases (MMP-3 and -13), inflammatory cytokines (IL-6), a disintegrin and metalloproteinase with thrombospondin motifs (ADAMTS-4 and -5), cyclooxygenase 2 (COX-2), prostaglandin E2 (PGE2) and free radicals [1]. Cartilage repair is mediated by anabolic growth factors including the transforming growth factor β (TGF-β), insulin-like growth factor 1 (IGF-1), bone morphogenetic proteins (BMPs) and fibroblast growth factors (FGF) [8]. The IGF-1, TGF-β and BMPs act in part by induction of the transcription factor SOX-9, a key regulator of mesenchymal chondrogenesis during embryologic development [9, 10]. No therapy is currently available to prevent the onset or progression of OA, and latest research has focused on disease-modifying drugs with the capacity not only to counteract the effects of pro-inflammatory cytokines but also to promote articular cartilage repair.

Anabolic-androgenic steroids (AAS) are synthetic derivatives of testosterone and were first introduced in the early 1930s. Since then, an attempt has been made to minimize the androgenic side effects by increasing their anabolic: androgenic ratio. Stanozolol has one of the highest anabolic: androgenic ratios, suggesting it has anabolic activity with minimal androgenic side effects [11]. Stanozolol has a wide range of applications in human medicine and has been used to treat rheumatoid arthritis [12, 13], hereditary angioedema [14], idiopathic osteonecrosis [15], postmenopausal osteoporosis [16, 17], muscle wastage and age-related sarcopenia [11], amongst others [18]. The main claims for stanozolol’s therapeutic effects are based on its anabolic activity on the musculoskeletal system and its potential to influence lean body mass, tissue repair, fibrinolysis, collagen synthesis and bone metabolism [11]. To our knowledge there are no human studies evaluating the effects of AAS on articular cartilage and there are no reports on the intra-articular use of stanozolol in human patients.

Intra-articular administration of stanozolol has recently been postulated as a treatment for OA. Studies investigating the clinical effects of the intra-articular use of stanozolol in horses have shown promising results in the ability of this drug to effectively manage lameness associated with OA and have suggested that stanozolol has the potential to stimulate cartilage repair [19, 20]. There is also anecdotal evidence of positive results obtained in equine clinical practice, including cases refractory to intra-articular glucocorticoids [21] and in the management of subchondral bone pain [22]. Furthermore, stanozolol has been used in an experimental animal model (sheep meniscectomy), demonstrating decreased osteophyte formation, subchondral bone reaction and synovial fibrosis, and articular cartilage regeneration [23]. However, there is limited evidence of the anti-inflammatory properties of stanozolol and how it affects articular chondrocytes in vitro. Previous data suggests that it reduces chondrocyte apoptosis by decreasing the production of nitric oxide in equine chondrocytes [24], enhances collagen synthesis though the upregulation of TGF-β in adult human dermal fibroblasts [25] and enhances the proliferation of cultured growth plate chondrocytes from pubertal female rats treated with GnRHa [26].

The objective of the current study is to evaluate the effect of stanozolol on chondrocyte viability and gene expression in normal chondrocytes and an in vitro inflammatory system of OA. We hypothesise that stanozolol will not have an effect on chondrocyte viability; will have a chondroprotective effect by reducing the expression of pro-inflammatory/catabolic genes (MMP-13, MMP-1, IL-6, ADAMTS4 and COX-2) in both normal and IL*-*1β treated chondrocytes; and will have an anabolic effect by increasing the expression of SOX9, COL2A1 and aggrecan in both normal and IL-1β treated chondrocytes.

## Methods

### Sample collection

Articular cartilage was aseptically harvested from the entire articular surface of macroscopically normal metacarpophalangeal joints of seven skeletally mature horses euthanized for reasons unrelated to this study, ages ranging from five to eleven years (mean 7.7 +/− SD 2.1). Samples were collected from an abattoir as a by-product of the agricultural industry and processed within 12 h of euthanasia. The Animals (Scientific Procedures) Act 1986, Schedule 2, does not define collection from these sources as scientific procedures and ethical approval was therefore not required.

### Tissue culture and cell viability

The chondrocytes were isolated as previously described [27] and the cell viability was determined by trypan blue exclusion test [28] for each horse. The chondrocytes were then plated as monolayers in Dulbecco’s modified eagles medium (DMEM) (Sigma-Aldrich, Dorset, UK), supplemented with 10% foetal calf serum (FCS), 100 units/ml penicillin, 100 mg/ml streptomycin (all from Invitrogen, Paisley, UK) and 500 ng/ml amphotericin B (BioWhittaker, Lonza, USA), at a concentration of 100,000 live cells/cm^2^ (total of 16 wells of a 24-well plate used per horse). The chondrocytes were incubated in a 5% CO_2_ humidified atmosphere at 37 °C for 48 h. The medium was changed and replaced with serum-free DMEM 24 h prior to exposure. Each horse was exposed to 4 different treatment groups (4 wells per group): (1) media only (negative control) (2) media + IL-1β (Recombinant Equine IL-1β, R&D Systems, Abingdon, UK) (10 ng/ml) (positive control) (3) media + IL*-*1β (10 ng/ml) + stanozolol (Sungate, ACME, Cavriago, Italy) (0.4 mg/ml) 4) media + stanozolol (0.4 mg/ml). The stanozolol concentration was extrapolated by using the clinically recommended dose of 5 mg per joint [20] and assuming the volume of a non-distended metacarpalphalangeal joint to be 12.5 ml [29]. After 24 h in culture conditions, cell viability was performed on one well per group.

### Real-time PCR

Total RNA was isolated separately from 3 wells per group using 0.5 ml of Tri Reagent (Sigma-Aldrich, Dorset, UK) per well and stored at -80 °C for later analysis. The RNA isolated from each well was processed individually and was purified by the acid guanidinium thiocyanate-phenol-chloroform extraction technique as previously described [30]. Total RNA concentrations and purity were determined spectrophotometrically (NanoDrop™ 1000 Spectrophotometer, Thermo Fisher Scientific, Waltham, US) and all the samples used presented a ratio of absorbance at 260 nm and 280 nm (A_260_/A_280_) between 1.8 and 2.0. cDNA was synthetized from 500 ng of RNA in a 29 μL reaction using Moloney Murine Leukemia Virus Reverse Transcriptase (M-MLV RT) and random hexamer oligonucleotides (both Promega, Southampton, UK). cDNA samples were individually diluted to a final concentration of 5 ng/μL and 5 μL aliquots of cDNA were amplified by reverse transcription polymerase chain reaction (RT-PCR) (ABI PRISM® 7500 Sequence Detection System, Applied Biosystems, Warrington, UK) in a 20 μL reaction volume using a SYBR Green PCR mastermix (Applied Biosystems, Warrington, UK). Glyceraldehyde 3-phosphate dehydrogenase (GAPDH) was used as the housekeeping gene and the relative expression of catabolic, anabolic and structural genes was analysed using the 2^–ΔΔC^_T_ method [31]. The fitness of GAPDH as a valid normalisation factor has been previously identified by us [32, 33]. All primers used were designed by Applied Biosystems Assays-by-Design and have been previously validated by our group. Primers for equine IL-6 and COX-2 had the following sequences: IL-6 Forward: CTG-CTC-CTG-GTG-ATG-GCT-AC, Reverse: CCG-AGG-ATG-TGT-ACT-TAA-TGT-GCT-G; COX-2 Forward: CAG-CAT-AAA-CTG-CGC-CTT-TTC, Reverse: AGG-CGG-GTA-GAT-CAT-TTC-CA. The primer sequences for GAPDH, SOX9, COL2A1, aggrecan, MMP-13 and -1, and ADAMTS4 have been previously reported [32, 34].

### Statistical analysis

Statistical analysis was performed using SPSS (IBM, Portsmouth, UK). Normality of the data was analysed using a Shapiro-Wilk test, and non-parametric data was LOG_10_ transformed prior to analysis. A general linear model was used for all analyses with Dunnet’s comparisons against the control group and pairwise comparisons with Bonferroni’s adjustment. Statistical significance was defined as *P* < 0.05.

## Results

### Stanozolol does not affect chondrocyte viability

There were no significant differences in cell viability between the different treatment groups.

### Stanozolol reduces catabolic gene expression

**Fig. 1:**
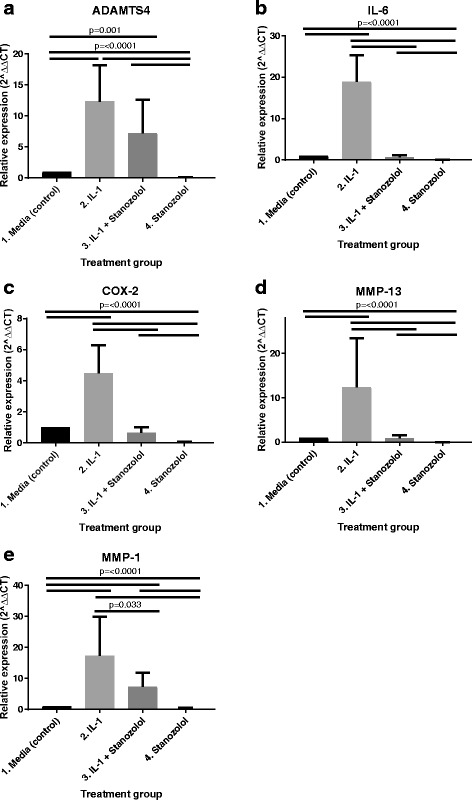
Effects of stanozolol on (**a**) ADAMTS4, (**b**) IL-6, (**c**) COX-2, (**d**) MMP-13 and (**e**) MMP-1 gene expression in normal and IL-1β-treated equine articular chondrocytes. Real-time-PCR analysis of the above genes mRNA in monolayer cultures exposed to media, IL-1β, IL-1β + stanozolol and stanozolol. GAPDH was used as the housekeeping gene and data are represented as relative expression using the 2^–ΔΔC^_T_ method. Data were evaluated using a general linear model with Dunnet’s comparisons against the control group and pairwise comparisons with Bonferroni’s adjustment (*n* = 7, three technical replicates per treatment group)

There was a statistically significant increase in the expression of MMP-13, MMP-1, IL-6, ADAMTS4 and COX-2 mRNA expression in chondrocytes treated with IL*-*1β only (group 2- positive control) compared to media only (group 1- negative control) (*P* < 0.001). Conversely, chondrocytes exposed to stanozolol only (group 4) had a significantly decreased expression of all the above catabolic genes compared to the negative control (group 1) (*P* < 0.001) (Fig. 1).

When comparing both groups with IL*-*1β treated chondrocytes, there was a significant downregulation of MMP-13 (*P* < 0.001), MMP-1 (*P* < 0.03), IL-6 (*P* < 0.001) and COX-2 (P < 0.001) expression in chondrocytes exposed to IL*-*1β and stanozolol (group 3) compared to chondrocytes exposed to IL*-*1β alone (group 2). ADAMTS4 gene expression was not significantly supressed in chondrocytes exposed to IL*-*1β and stanozolol (group 3) compared to chondrocytes exposed to IL*-*1β only (group 2) (Fig. 1).

### Stanazolol reduces COL2A1 gene expression

**Fig. 2:**
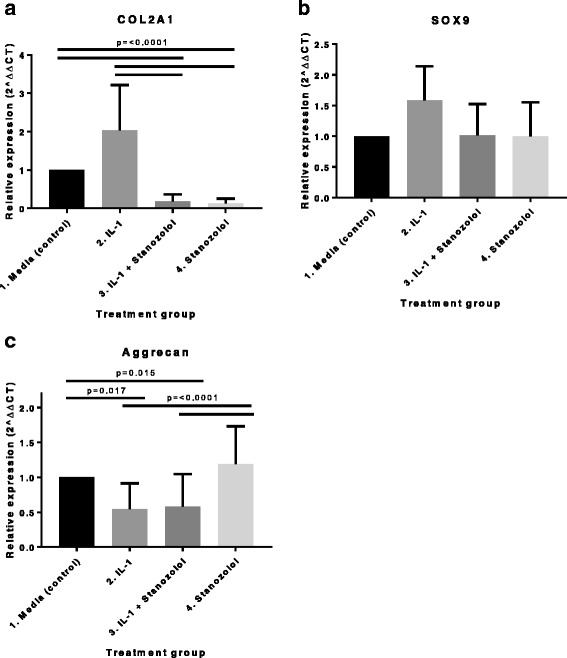
Effect of stanozolol on (**a**) COL2A1, (**b**) SOX-9 and (**c**) aggrecan gene expression in normal and IL-1β-treated equine articular chondrocytes. Real-time-PCR analysis of the above genes mRNA in monolayer cultures exposed to media, IL-1β, IL-1β + stanozolol and stanozolol. GAPDH was used as the housekeeping gene and data are represented as relative expression using the 2^–ΔΔC^_T_ method. Data were evaluated using a general linear model with Dunnet’s comparisons against the control group and pairwise comparisons with Bonferroni’s adjustment (*n* = 7, three technical replicates per treatment group)

There was no statistically significant difference in SOX9 gene expression between treatment groups. COL2A1 gene expression was significantly supressed in both groups treated with stanozolol (groups 3 and 4) compared to both groups not treated with stanozolol (groups 1 and 2). Aggrecan gene expression was significantly supressed in both groups treated with IL*-*1β (groups 2 and 3) compared to both groups not treated with IL*-*1β (groups 1 and 4). No statistically significant difference was seen on COL2A1 gene expression between groups 1 and 2 or 3 and 4, or on aggrecan expression between groups 1 and 4 or 2 and 3 (Fig. 2).

## Discussion

In OA the balance between anabolic and catabolic activities is compromised and cartilage degeneration prevails over the capacity of repair. In the present study, treatment with stanozolol was investigated in both normal and IL-1β stimulated chondrocytes. In accordance with previous reports [35–37] exposure to IL-1β induced a catabolic response on equine articular chondrocytes and increased the gene expression of MMP-13, MMP-1, IL-6, ADAMTS4 and COX-2. Addition of stanozolol to IL-1β stimulated chondrocytes counteracted the catabolic effects of IL-1β and downregulated the expression of MMP-13, MMP-1, IL-6, and COX-2. Similarly, corticosteroids have been demonstrated to inhibit MMP transcription [38–42] and downregulate the expression of COX-2 [40–42] in cytokine-treated chondrocytes. In the present study, stanozolol supressed ADAMTS4 gene expression in normal chondrocytes compared to control but did not counteract the upregulation induced by IL-1β. This is in contrast with observations in corticosteroids studies [39, 40].

Further analysis of our results showed that when normal chondrocytes were treated with stanozolol there was a significant decrease in the expression of all the catabolic genes tested (MMP-13, MMP-1, IL-6, ADAMTS4 and COX-2) compared to control. Again, similar results have been obtained in in vitro models studying the effects of corticosteroids on articular cartilage [38, 39]. Knych et al. [43] recently evaluated changes in synovial fluid gene expression following in vivo administration of triamcinolone in healthy horses undergoing a standardized exercise programme. In this study they found no significant differences in COX-2 and MMP-3 gene expression between baseline and the triamcinolone treatment group. These results suggest a protective and stronger anti-inflammatory response after intra-articular administration of stanozolol compared to triamcinolone; however, different experimental models limit direct comparisons between studies.

Contrary to our hypothesis, stanozolol did not enhance the expression of SOX-9, COL2A1 or aggrecan. The pathway through which anabolic growth factors promote upregulation of chondrocyte specific genes remains largely unclear. The transcription factor SOX9 has elicited much interest as it is co-expressed with type II collagen in mouse fetal chondrocytes and is thought to be involved in chondrocyte differentiation and cartilage formation during development [44]. SOX9 has been shown to mediate the expression of many genes encoding cartilage extracellular matrix proteins, including COL2A1 and aggrecan [9, 45, 46]. Kolettas et al. [9] investigated the effects of IGF-1 and IL-1 on chondrocyte survival and phenotype and reported an antagonist effect. IL-1 supressed the expression of chondrocyte-specific genes (collagen types II and IX, aggrecan, biglycan and link protein) and downregulated SOX-9; and IGF-1 upregulated SOX-9, relieved the IL-1 induced inhibition of chondrocyte-specific genes and enhanced chondrocyte survival. The authors therefore suggested that IGF-1 and IL-1 modulate chondrocyte survival and differentiation through changes in SOX-9 gene expression. Further studies have challenged this concept and showed a negative correlation between COL2A1 and SOX9 gene expression. In human adult articular chondrocytes, IL-1 had no significant influence on SOX9 mRNA expression whereas COL2A1 was significantly down-regulated. The same experiment also demonstrated that IGF-I up-regulated COL2A1 expression, but not SOX9 [47]. In our study, treatment with IL-1 and stanozolol, alone or in combination, did not alter SOX-9 gene expression in comparison to control. IL-1 did not downregulate SOX-9 as previously reported, had no effect on COL2A1 and downregulated aggrecan expression. Furthermore, treatment with stanozolol supressed COL2A1 without affecting SOX-9 gene expression. Our results suggest that COL2A1 and aggrecan gene expression are not correlated with the expression of SOX9 in equine articular chondrocytes and that treatment with stanozolol does not elicit an anabolic response through the upregulation of SOX-9, COL2A1 or aggrecan.

Both COL2A1 and aggrecan are important structural genes essential for cartilage integrity and formation. Stanozolol has been shown to increase collagen synthesis in cultures of human dermal fibroblasts [25]. The decrease in COL2A1 levels, which we have observed when chondrocytes were treated with stanozolol, goes against what has been reported in human dermal fibroblasts, and is similar to that reported by Knych et al. [43] in synovial fluid and Richardson and Dodge [38] in articular chondrocytes following treatment with corticosteroids. In the present study, aggrecan expression was downregulated by IL-1β and treatment with stanozolol did not ameliorate the IL-1 inhibition of aggrecan, which is also in accordance with what has been reported with corticosteroids [38]. Furthermore, the downregulation of COL2A1 induced by stanozolol in this study warrants careful consideration of its intra-articular use as it may suggest deleterious effects on cartilage. A limitation of our study is the use of a short-term in vitro system and different results may have been obtained with longer exposure times or with repeat exposures mimicking what has been described in clinical trials. Additionally, this study used chondrocytes and not cartilage explants. However, we used freshly isolated chondrocytes plated at high density in order to reduce dedifferentiation of chondrocytes in cell culture which can results in an altered phenotype. Future studies using cartilage explants are needed.

The similarities between the anti-catabolic effects of stanozolol and corticosteroids might be associated with the previously reported affinity of AAS for the glucocorticoid receptors [48]. Alternative pathways through which AAS might be able to exert an anabolic effect on articular tissues merit further investigation; and a plausible explanation for the lack of anabolic activity in our in vitro system might be related to a single and short-lived exposure time. Further studies investigating the systemic effects of a low intra-articular dose of stanozolol and its local effects on articular tissues with naturally-occurring OA are warranted. The use of AAS is banned by the main organizations overseeing drug regulation in sport, therefore its use should only be considered in non-competing individuals.

## Conclusions

Stanozolol has been described as having disease-modifying activity by attenuating the degenerative response in osteoarthritic cartilage in in vivo studies. The results of our in vitro study support the hypothesis that stanozolol has an anti-inflammatory effect and is effective at inhibiting the production of pro-inflammatory mediators in both normal and IL-1 treated chondrocytes. However, in our in vitro system there is no evidence of stanozolol having an anabolic effect through upregulation of SOX-9, COL2A1 and aggrecan gene expression.

## Abbreviations

AAS: Anabolic-androgenic steroids
ADAMTS: Disintegrin and metalloproteinase with thrombospondin motifs
BMPs: Bone morphogenetic proteins
cDNA: Complimentary deoxyribonucleic acid
CO_2_: Carbon dioxide
COL2A1: Collagen type 2 alpha 1
COX-2: Cyclooxygenase-2
DMEM: Dulbecco’s modified eagles medium
FCS: Foetal calf serum
FGF: Fibroblast growth factor
GAPDH: Glyceraldehyde 3-phosphate dehydrogenase
GnRHa: Gonadotropin-releasing hormone agonist
IGF-1: Insulin-like growth factor 1
IL-1β: Interleukin-1β
IL-6: Interleukin 6
M-MLV RT: Moloney Murine Leukemia Virus Reverse Transcriptase
MMP: Matrix metalloproteinase
mRNA: Messenger ribonucleic acid
OA: Osteoarthritis
PGE2: Prostaglandin E2
RT-PCR: Reverse transcription polymerase chain reaction
SOX-9: Transcripton gactor SOX-9
TGF-β: Transforming growth factor β
